# Establishment of Level a In Vitro–In Vivo Correlation (IVIVC) via Extended DoE-IVIVC Model: A Donepezil Case Study

**DOI:** 10.3390/pharmaceutics14061226

**Published:** 2022-06-09

**Authors:** Da Young Lee, Soyoung Shin, Tae Hwan Kim, Beom Soo Shin

**Affiliations:** 1 School of Pharmacy, Sungkyunkwan University, 2066 Seobu-ro, Jangan-gu, Suwon 16419, Korea; dayoung717@skku.edu; 2 College of Pharmacy, Wonkwang University, 460 Iksan-daero, Iksan 54538, Korea; shins@wku.ac.kr; 3 College of Pharmacy, Daegu Catholic University, 13-13 Hayang-ro, Hayang-eup, Gyeongsan 38430, Korea; thkim@cu.ac.kr

**Keywords:** level A IVIVC, donepezil, mixture design, population pharmacokinetics, sustained release, prediction of in vivo pharmacokinetics

## Abstract

This study aimed to establish an extended design of experiment (DoE)-in vitro in vivo correlation (IVIVC) model that defines the relationship between formulation composition, in vitro dissolution, and in vivo pharmacokinetics. Fourteen sustained-release (SR) tablets of a model drug, donepezil, were designed by applying a mixture design of DoE and prepared by the wet granulation method. The in vitro dissolution patterns of donepezil SR tablets were described by Michaelis–Menten kinetics. The mathematical relationship describing the effects of SR tablet compositions on the in vitro dissolution parameter, i.e., the in vitro maximum rate of release (V_max_), was derived. The predictability of the derived DoE model was validated by an additional five SR tablets with a mean prediction error (PE%) of less than 3.50% for in vitro V_max_. The pharmacokinetics of three types of donepezil SR and the immediate-release (IR) tablets was assessed in Beagle dogs following oral administration (*n* = 3, each). Based on the plasma concentration-time profile, a population pharmacokinetic model was developed, and the in vivo dissolution of SR tablets, represented by in vivo V_max_, was estimated. By correlating the in vitro and in vivo V_max_, level A IVIVC was established. Finally, the extended DoE-IVIVC model was developed by integrating the DoE equation and IVIVC into the population pharmacokinetic model. The extended DoE-IVIVC model allowed one to predict the maximum plasma concentration (C_max_) and the area under the plasma concentration-time curve (AUC) of donepezil SR tablets with PE% less than 10.30% and 5.19%, respectively, by their formulation composition as an input. The present extended DoE-IVIVC model may provide a valuable tool to predict the effect of formulation changes on in vivo pharmacokinetic behavior, leading to the more efficient development of SR formulations. The application of the present modeling approaches to develop other forms of drug formulation may be of interest for future studies.

## 1. Introduction

In vitro in vivo correlation (IVIVC) has been of great interest as a promising approach for the successful development of new dosage forms. The US Food and Drug Administration (FDA) has defined IVIVC as “a predictive mathematical model describing the relationship between an in vitro property of an extended-release (ER) dosage form and a relevant in vivo response” [1]. Thus, IVIVC is intended to predict the in vivo performance of a drug product based on its in vitro drug release profiles by correlating the in vitro dissolution and in vivo pharmacokinetics. As a significant amount of time and cost is required to obtain the optimal in vivo pharmacokinetics during the formulation development process, IVIVC may provide an engaging strategy to facilitate the successful development of a new formulation. IVIVC can also support regulatory applications for bioequivalence based on in vitro dissolution data or clinically relevant in vitro specifications [2,3].

Nonetheless, the application of IVIVC has been primarily limited to determining the inter-relationship between the in vitro properties and in vivo responses of the dosage forms once they are prepared. Hence, although the establishment of IVIVC could propose the in vitro release patterns to accomplish a target in vivo response, the formulation design to obtain the optimal in vitro and in vivo properties is still primarily empirical. The extension of IVIVC to the earlier phase of formulation design that could quantitatively evaluate the impact of formulation composition on the in vivo pharmacokinetics has been pursued just recently [4,5]. 

The design of experiment (DoE) is a statistical method to determine the relationship between the formulation factors with the in vitro properties of a drug product. Therefore, it was postulated that the impact of formulation changes on the in vivo pharmacokinetics might be predicted by combining the DoE and IVIVC for the development of an optimal sustained-release (SR) tablet. If the in vivo pharmacokinetics can be predicted in the early stage of formulation development, we could save a lot of time and cost for the development of SR formulations. Therefore, this study aimed to demonstrate the potential utility of the extended DoE-IVIVC model to predict the pharmacokinetics from the formulation composition by using donepezil as a model drug. 

The model drug, donepezil, which has been marketed under the trade name Aricept^®^, is a medication used for the treatment of mild, moderate, and severe Alzheimer’s disease. As a reversible acetylcholinesterase inhibitor with high potency and selectivity for centrally active cholinesterase [6], donepezil has been prescribed for the symptomatic treatment of Alzheimer’s disease. However, the difficulty in swallowing as well as the gastrointestinal side effects of donepezil tablets are often associated with the patients’ incompliance, leading to poor therapeutic efficacy [7]. Moreover, an initial sharp increase in the blood concentration after administration of donepezil immediate-release (IR) formulations could lead to unwanted cholinergic side effects such as nightmares, insomnia, anxiety, and nausea [8]. Thus, an SR dosage form of donepezil is highly desirable for patients with Alzheimer’s disease. Physicochemical examination indicated that donepezil is classified as a biopharmaceutical classification system (BCS) class IIc drug, which is a neutral drug with low solubility and high permeability [9]. 

In this study, the potential of the extended DoE-IVIVC model to predict the impact of formulation changes on the in vivo pharmacokinetics has been demonstrated for donepezil SR tablets. The relationship between the formulation compositions and the in vitro dissolution response has been determined by the DoE model. The in vitro dissolution and the population pharmacokinetic model-estimated in vivo dissolution were correlated to develop IVIVC. Finally, DoE and IVIVC have been integrated into the population pharmacokinetic model that correlates the formulation composition, in vitro dissolution, and in vivo pharmacokinetics, establishing the extended DoE-IVIVC model.

## 2. Materials and Methods

### 2.1. Materials

Donepezil hydrochloride was purchased from Sinoway Industrial Co., Ltd. (Xiamen, China). Donepezil-D_7_ was obtained from Toronto Research Chemicals Inc. (Toronto, ON, Canada). Aricept^®^ 5 mg was from Daewoong Pharmaceutical Co., Ltd. (Seoul, Korea). Hydroxypropyl methylcellulose (HPMC) 2208–100 cps and HPMC 2208–4000 cps were purchased from Shin-Etsu Chemical Co., Ltd. (Tokyo, Japan). Lactose and magnesium stearate were purchased from Whawon Pharm. Co., Ltd. (Seoul, Korea) and Faci Asia Pacific Pte Ltd. (Jurong Island, Singapore), respectively. Acetic acid and formic acid were obtained from Sigma-Aldrich Co. (St. Louis, MO, USA). Hydrochloric acid, potassium dihydrogen phosphate, and ethanol were obtained from Merck Co. (Darmstadt, Germany). Sodium hydroxide and sodium chloride were purchased from Samchun Chemical Co., Ltd. (Seoul, Korea). HPLC-grade acetonitrile and water were purchased from J.T. Baker Co. (Philipsburg, NJ, USA).

### 2.2. Quantitative Analysis of Donepezil

#### 2.2.1. HPLC

The high-performance liquid chromatography (HPLC) analysis of donepezil was performed by a Waters Alliance 2695 separation module coupled with a Waters 2996 photodiode array detector (Waters, Milford, MA, USA). Donepezil in the dissolution medium samples was separated on an Eclipse plus C18 column (2.1 × 150 mm, i.d., 5 µm, Agilent Technologies, Santa Clara, CA, USA) with Eclipse plus C18 (2.1 × 5 mm, i.d., 1.8 µm, Agilent Technologies). An isocratic solvent system consisting of acetonitrile, methanol, and 10 mM ammonium phosphate buffer (pH 2.6 modified by the addition of phosphoric acid) (30:20:50 *v*/*v*/*v* %) was used as the mobile phase at a flow rate of 0.2 mL/min. The column oven temperature was 40 °C, and the total run time was 4 min. The sample injection volume was 20 µL, and donepezil was detected at 270 nm. 

#### 2.2.2. LC–MS/MS 

The liquid chromatography–tandem mass spectrometry (LC–MS/MS) analysis of donepezil was performed by an Agilent 6430 triple-quadrupole mass spectrometer coupled with an Agilent 1200 HPLC (Agilent Technologies). A stable isotope-labeled donepezil-D_7_ was used as an internal standard (IS). Acetonitrile was used as a protein precipitation agent to extract donepezil from the plasma samples. Donepezil was separated on an Eclipse plus C18 column (2.1 × 150 mm, i.d., 5 µm, Agilent Technologies) with an Eclipse plus C18 (2.1 × 5 mm, i.d., 1.8 µm, Agilent Technologies). An isocratic solvent system consisting of 0.1% aqueous formic acid and acetonitrile (70:30 *v/v* %) was used as the mobile phase. The flow rate of the mobile phase was maintained at 0.3 mL/min. The column oven temperature was 40 °C, and the total run time was 5 min.

The mobile phase was introduced into the mass spectrometer via the electrospray ionization (ESI) source operating in the positive mode under multiple reaction monitoring (MRM) mode. Nitrogen was utilized as the nebulizer gas at 35 psi with a flow rate of 10 L/min and a temperature of 350 °C. The selected MRM transition was 380.1 → 91.1 for donepezil and 387.1 → 98.1 for donepezil-D_7_ (IS). The lower limit of quantification (LLOQ) of donepezil in the dog plasma was 0.05 ng/mL. The LC–MS/MS method was fully validated according to the FDA guidance [10]. 

### 2.3. Development of DoE Model

#### 2.3.1. Mixture Design of Donepezil SR Tablets

**Figure 1:**
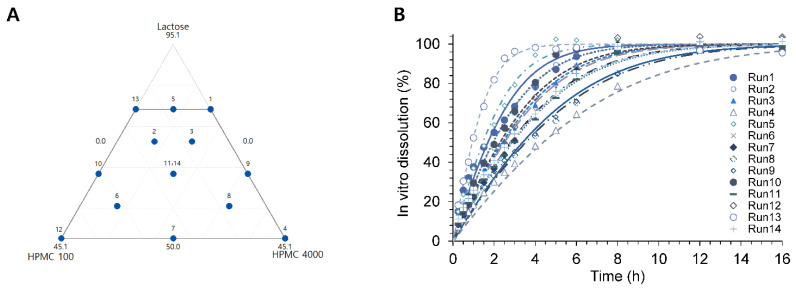
Fourteen experimental points of donepezil SR tablets on the simplex design plot (**A**) and the respective in vitro dissolution profiles at pH 1.2 (**B**).

Various compositions of donepezil SR tablets with different drug release rates were designed by the mixture design by Minitab^®^ 18 (Minitab, LLC, State College, PA, USA) [5]. HPMC 2208–100 cps and HPMC 2208–4000 cps were employed as drug release rate modifiers, and lactose was used as a diluent. Lactose (x_1_, 50.0–80.0%), HPMC 2208–100 cps (x_2_, 0.0–45.1%), and HPMC 2208–4000 cps (x_3_, 0.0–45.1%) were designated as control factors. All SR tablets have fixed amounts of donepezil hydrochloride (3.91%, 5 mg as donepezil) and magnesium stearate (0.99%) in common. According to the mixture experimental design, a total of 14 SR tablets, including two replicates of the center point (Run 11 and 14), were randomly arranged (Table 1, Figure 1A). 

#### 2.3.2. Preparation of Donepezil SR Tablets

Fourteen donepezil SR tablets designed by the mixture design were prepared by a wet granulation method, as described previously [5,11], with modification. Donepezil hydrochloride was mixed with lactose, HPMC 2208–100 cps, and 4000 cps in a plastic bag for 10 min, followed by the addition of the binder solution (60% ethanol). The mixture was kneaded and passed through the 20-mesh sieve, followed by drying in an oven at 50 °C for 60 min. After drying, the granules were additionally sieved through the 20-mesh sieve, and magnesium stearate (0.990%) was added. The lubricated resultant mixture was weighed and compressed (1 ton) by a hydraulic tablet press (Carver, Inc., Wabash, IN, USA) with a round-shaped punch (diameter: 7.0 mm). 

#### 2.3.3. In Vitro Dissolution

In vitro dissolution studies were performed by the paddle method (USP Apparatus 2 guideline) using the Distek Dissolution System 2500 coupled with the Evolution Dissolution Sampler 4300 (North Brunswick, NJ, USA). The dissolution media was 0.1 N HCl (pH 1.2) maintained at 37 ± 0.5 °C. The paddle stirring speed was fixed at 100 rpm. The aliquots (2 mL) of samples were collected by an auto-sampler at predetermined time intervals and filtered using a 45 µm polyethylene syringe filter (Distek, North Brunswick, NJ, USA). Obtained medium samples were immediately analyzed by the HPLC assay.

The differential equation for the amount of undissolved donepezil in the dissolution medium (X_Tablet in vitro_) was

(1) $$\frac{{\mathrm{dX}}_{\mathrm{Tablet}\cdot \mathrm{in}\;\mathrm{vitro}}}{\mathrm{dt}}=-\frac{V_{\max\cdot \mathrm{in}\;\mathrm{vitro}}}{{\mathrm{AM}}_{50\cdot \mathrm{in}\;\mathrm{vitro}}+X_{\mathrm{Tablet}\cdot \mathrm{in vitro}}}\cdot X_{\mathrm{Tablet}\cdot \mathrm{in vitro}}$$

where V_max·in vitro_ and AM_50·in vitro_ represent the in vitro maximum rate of donepezil release and the amount of donepezil at which the dissolution rate is half of V_max·in vitro_, respectively. 

To correct the effect of different dose, both V_max·in vitro_ and AM_50·in vitro_ were normalized by the donepezil dose in the tablet. The dissolution data were fitted to the model using the Berkeley Madonna (version 8.3.18, Berkeley Madonna, Albany, CA, USA). 

#### 2.3.4. Development of the DoE Model

The mixture design module in Minitab^®^ 18 (Minitab Inc., State College, PA, USA) was used to examine the effect of control factors (x_1_, x_2_, and x_3_) on the response factor (y), V_max·in vitro_. The response was assumed to only depend on the proportions of the components (x_1_, x_2_, and x_3_) of the mixture design. Mixture regression was used as a model-fitting method. Finally, the DoE model in which all variables have *p*-values less than the specified α = 0.05 was obtained.

#### 2.3.5. Validation of the DoE Model

**Figure 2:**
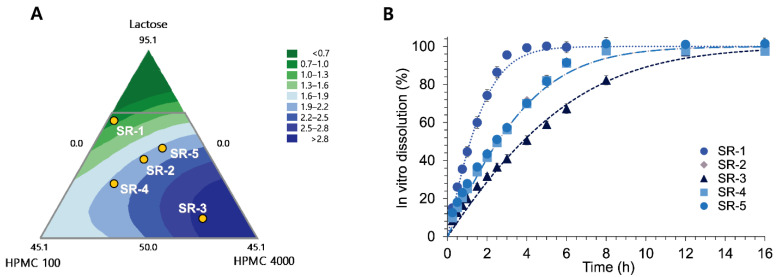
Five experimental points of donepezil SR tablets for the DoE validation on the contour plot (**A**), and their respective in vitro dissolution profiles at pH 1.2 (**B**).

The DoE model was evaluated by external validation by an additional five SR tablets of donepezil (SR-1~SR-5). The five SR tablets for external validation were intended to have fast (SR-1), medium (SR-2), and slow (SR-3) drug release rates (Figure 2A). SR-4 and SR-5 tablets were selected from the same region of the contour plot as SR-2 to have the same dissolution rate but different formulation compositions. After the SR tablets for validation were produced as indicated, the respective in vitro dissolution parameters, V_max·in vitro,_ were determined identically to the fourteen SR tablets for model development. The observed V_max·in vitro_ was compared to the DoE model-predicted V_max·in vitro,_ and the prediction error (PE%) was calculated.

(2) $$\mathrm{PE}\%=\frac{|\mathrm{Predicted}-\mathrm{Observed}|}{\mathrm{Observed}}\times 100$$

### 2.4. Beagle Dog Study

Among the five SR tablets for external validation, three formulations (SR-1, SR-2, and SR-3) were selected to evaluate in vivo pharmacokinetics in Beagle dogs for the development of DoE-IVIVC model. All animals received care by the ethics committee for the treatment of laboratory animals at KNOTUS CO., Ltd. (Guri, Korea) (KNOTUS IACUC 19-KE-575).

A total of 12 dogs were randomly allocated into 4 groups (*n* = 3, each) and fasted overnight before drug administration. An IR tablet containing 4.562 mg of donepezil (Aricept^®^) and three SR tablets containing 5 mg of donepezil (SR-1, SR-2, and SR-3) were orally administered to Beagle dogs (11–12 months, body weight 8.3–12.8 kg). Blood samples (3 mL) were collected via the cephalic vein into a heparinized (5 IU/mL) tube following the administration of the tablets at 0, 0.25, 0.5, 0.75, 1, 1.5, 2, 2.5, 3, 3.5, 4, 4.5, 5, 5.5, 6, 8, 12, 24, 36, and 48 h. After blood samples were centrifuged at 4000× *g* for 10 min, the obtained plasma samples were immediately frozen and stored at −20 °C until analysis. 

The observed plasma concentration vs. time profiles after the oral administration of IR or SR tablets of donepezil were analyzed by noncompartmental analysis. The relative bioavailability (%) was calculated by the ratio of dose-normalized area under the plasma concentration-time curve from time zero to the last observation time point (AUC_last_) of the SR tablet to the IR reference tablet. 

### 2.5. Development of DoE-IVIVC Model

#### 2.5.1. Population Pharmacokinetic Model

The DoE-IVIVC model was developed as described previously [5], with modification. To establish IVIVC, a population pharmacokinetic model was developed to estimate in vivo dissolution from SR tablets in the gastrointestinal tract. While the drug release from the IR tablet was assumed to be instantaneous, the drug dissolution process from SR tablets, i.e., from the tablet compartment to the gut compartment, was described by the Michaelis–Menten kinetics. The absorption process, i.e., from the gut compartment to the central compartment, was described by first-order kinetics with K_a_. The differential equations for the amounts of the undissolved drug in the tablet compartment (X_Tablet·in vivo_) and the dissolved drug in the gut compartment (X_gut_) were written as below:

(3) $$\frac{{\mathrm{dX}}_{\mathrm{Tablet}\cdot \mathrm{in vivo}}}{\mathrm{dt}}=-\frac{V_{\max\cdot \mathrm{in vivo}}}{{\mathrm{AM}}_{50\cdot \mathrm{in vivo}}+X_{\mathrm{Tablet}\cdot \mathrm{in vivo}}}\;\cdot X_{\mathrm{Tablet}\cdot \mathrm{in vivo}}$$

(4) $$\frac{{\mathrm{dX}}_{\mathrm{gut}}}{\mathrm{dt}}={\;F}_{\mathrm{abs}}\cdot \frac{V_{\max\cdot \mathrm{in vivo}}}{{\mathrm{AM}}_{50\cdot \mathrm{in vivo}}+X_{\mathrm{Tablet}\;\cdot \mathrm{in vivo}}}\;\cdot X_{\mathrm{Tablet}\;\cdot \mathrm{in vivo}}-K_{a}\cdot X_{\mathrm{gut}}$$

where V_max·in vivo_ is the maximum rate of dissolution from the donepezil tablet in the gastrointestinal tract, and AM_50·in vivo_ is the amount of donepezil at which half of the maximum dissolution rate (V_max·in vivo_) is achieved. To correct the effect of different doses, both V_max·in vivo_ and AM_50·in vivo_ were normalized by the donepezil dose in the tablet. F_abs_ is the absorbable fraction of donepezil over time described by

(5) $$F_{\mathrm{abs}}=1-\left(\;\frac{{\mathrm{Time}}^{\mathrm{Hill}}}{T_{\mathrm{abs}}{}^{\mathrm{Hill}}+{\mathrm{Time}}^{\mathrm{Hill}}}\;\right)\;$$

Based on this equation, the declining pattern of F_abs_ was described by T_abs,_ the time associated with the maximal changes in F_abs_, and the Hill coefficient. Time is the time after the oral administration of donepezil tablets. The change in F_abs_ over time allowed the model to accommodate a complex absorption pattern, as demonstrated by previous studies [4,11].

A two-compartment model was used to describe the pharmacokinetics of donepezil. The differential equations for the donepezil amounts in the central (X_1_) and peripheral (X_2_) compartments were written as follows:

(6) $$\frac{{\mathrm{dX}}_{1}}{\mathrm{dt}}={\;K}_{a}\cdot X_{\mathrm{gut}}-\mathrm{CL}\cdot C_{1}-\mathrm{CLD}\cdot C_{1}+\mathrm{CLD}\cdot C_{2}$$

(7) $$\frac{{\mathrm{dX}}_{2}}{\mathrm{dt}}=\;\mathrm{CLD}\cdot C_{1}-\mathrm{CLD}\cdot C_{2}$$

CL is the systemic clearance, while CLD is the distribution clearances. C_1_ and C_2_ are the donepezil concentrations in the central and peripheral compartments, respectively. The plasma concentration-time data were simultaneously fitted to the population PK model using S-ADAPT (version 1.57, Biomedical Simulations Resource, Los Angeles, CA, USA) to estimate model parameters, including in vivo dissolution parameter, V_max·in vivo_. Between-subject variability (BSV) was estimated by the exponential parameter variability model.

The in vivo dissolution profiles of three donepezil SR tablets were estimated by the developed population PK model. The dissolution parameter, V_max·in vivo,_ for each SR tablet was then correlated with the respective in vitro dissolution parameter V_max·in vitro_ via regression analysis using SigmaPlot (version 12.0, Systat Software, Inc., San Jose, CA, USA). 

#### 2.5.2. DoE-IVIVC Model

The DoE model that describes the effect of formulation composition on in vitro dissolution and the IVIVC equation to correlate in vitro and in vivo V_max_ were finally introduced to the population pharmacokinetics model, establishing the DoE-IVIVC model. By connecting the two models, the DoE-IVIVC was developed, which could predict the plasma concentration-time profile from the formulation composition, and vice versa.

The predictability of the developed DoE-IVIVC model was validated by comparing the model-predicted pharmacokinetic parameters, i.e., C_max_ and AUC_last_, to the observed values. Based on the developed model, a thousand Monte Carlo simulations were performed using Berkeley Madonna (version 8.3.18). C_max_ and AUC_last_ were predicted from individual plasma concentration-time profiles and compared with the observed values. By calculating the prediction error (PE%), the model’s predictability was assessed according to the FDA guidance for conventional IVIVC [1].

### 2.6. Statistical Analyses

The data were statistically tested by one-way analysis of variance (ANOVA) followed by Tukey post hoc test. The statistical significance level was set at *p* < 0.05. Statistical analyses were performed using SPSS (IBM, Armonk, NY, USA).

## 3. Results

### 3.1. DoE for Donepezil SR Formulations

#### 3.1.1. Mixture Design of Donepezil SR Formulations

The formulation compositions of fourteen donepezil SR tablets designed by DoE and their experimentally determined 1/V_max·in vitro_ values were presented in Table 1. The formulation compositions of 14 donepezil SR tablets in the experimental region are depicted in Figure 1A, and the respective in vitro dissolution profiles are shown in Figure 1B. The observed Run orders 11 and 14 are two replicates of the center point. 

As shown in Table 1, the fractional compositions of HPMC 2208–100 cps and HPMC 2208–4000 cps varied from 0 to 45.1% per tablet to evaluate the effect of the amount and viscosity of the hydrophilic polymers on dissolution rates. The amount of lactose ranged from 50 to 80%. The sum of control factors, i.e., lactose, HPMC 2208–100 cps, and HPMC 2208–4000 cps, was fixed to 95.1%. All SR tablets contained fixed amounts of donepezil hydrochloride (3.910%) and magnesium stearate (0.990%). The in vitro dissolution profiles of donepezil from SR tablets (Figure 1B) were best described by Michaelis–Menten kinetics. As the amount of HPMC 2208–100 cps and HPMC 2208–4000 cps in the SR tablet increased from 0 to 45.1% per tablet, 1/V_max·in vitro_ increased from 0.729 to 3.448 h, indicating slower dissolution. 

The resulting mathematical equation to describe the effect of control factors, i.e., x_1_ (lactose), x_2_ (HPMC 2208–100 cps), and x_3_ (HPMC 2208–4000 cps), on the dissolution parameter, 1/V_max·in vitro_, in coded terms is as follows:

(8) $$1/V_{\max\cdot \mathrm{in vitro}}\;=\;-0.0082655\cdot x_{1}-0.0190053\cdot x_{2}+0.0080097\cdot x_{3}+0.0013596\cdot x_{1}x_{2}+0.0015589\cdot x_{1}x_{3}\;-0.0046015\cdot x_{2}x_{3}+0.000076\cdot x_{1}x_{2}x_{3}-0.0000113\cdot x_{2}x_{3}\left(x_{2}-x_{3}\right)$$

The contour plots between the independent variables (lactose, HPMC 2208–100 cps, and HPMC 2208–4000 cps) for 1/V_max·in vitro_ are shown in Figure 2A. The goodness of fit is represented by statistics, including the actual model R^2^, the adjusted R^2^, and the predicted R^2^, which were 99.79%, 99.54%, and 96.50%, respectively.

#### 3.1.2. Validation of DoE Model

External validation of the developed DoE model was performed. Five compositions of donepezil (SR-1–SR-5) were selected from the contour plot for validation (Figure 2A). The compositions (*w*/*w*%) of donepezil SR tablets for external validation are shown in Table 2. While SR-1, SR-2, and SR-3 were selected from the different regions of the contour plot to have the fast, medium, and slow dissolution rates, respectively, SR-4 and SR-5 were selected from the same area as SR-2. Thus, SR-4 and SR-5 were intended to have identical medium dissolution rates to SR-2 despite the different formulation compositions. 

Figure 2B shows the in vitro dissolution patterns of SR-1–SR-5. Consistent with the prediction from their formulation compositions via the DoE model, SR-1 showed the most rapid dissolution while SR-3 showed the slowest dissolution. The drug-release patterns of SR-2, SR-4, and SR-5 overlapped to have moderate dissolution rates, represented by the time to achieve 80% dissolution (T_80_) of 4.78 ± 0.04 h, 4.84 ± 0.06 h, and 4.88 ± 0.24 h, respectively, suggesting comparable drug release rates. T_80_ of SR-1 (2.24 ± 0.12 h) and SR-3 (8.35 ± 0.31 h) were significantly different from those of SR-2, SR-4, and SR-5. The experimentally determined 1/V_max·in vitro_ compared to the DoE model-predicted 1/V_max·in vitro_ from their formulation compositions are shown in Table 2. The PE% of donepezil SR tablets ranged from 0.56 to 3.50%, with an average PE% of 2.40%.

### 3.2. Pharmacokinetics of Donepezil in Beagle Dogs

**Figure 3:**
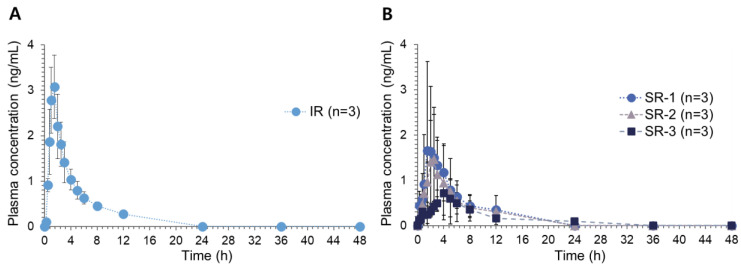
The average plasma concentration-time profiles of donepezil after the oral administration of (**A**) the reference IR tablet (as donepezil 4.562 mg, *n* = 3) and (**B**) SR-1, SR-2, and SR-3 (as donepezil 5 mg) (*n* = 3, each) in Beagle dogs.

The pharmacokinetics of donepezil were determined following oral administration of the reference IR tablet and three SR tablets, i.e., SR-1, SR-2, and SR-3, in Beagle dogs. For oral dosage forms, the test formulation needs to be evaluated in large animals with the ability to ingest the same dosage forms intended for clinical use. The gastrointestinal physiology of Beagle dogs, including gastric pH and gastric residence time, is also comparable to humans [12,13]. Beagle dogs were therefore selected as the animal model for the present study to establish IVIVC. In addition, dogs exhibit age-dependent cognitive decline and cholinergic hypofunction similar to patients with Alzheimer’s disease [14], which makes dogs a helpful model in the screening of therapeutics for Alzheimer’s disease. The pharmacological activities of cholinesterase inhibitors such as donepezil have been well demonstrated in the canine model as well [14]. Figure 3 shows the average plasma concentration-time profiles of donepezil following the oral administration of an IR reference tablet (donepezil 4.562 mg per tablet, *n* = 3) and three SR tablets (donepezil 5 mg per tablet, *n* = 3). The non-compartmental pharmacokinetic parameters of donepezil are summarized in Table 3. 

Following the oral administration of the IR tablet, donepezil was rapidly absorbed to achieve C_max_ in 1.0–1.5 h. After reaching C_max_, donepezil plasma concentrations showed a bi-exponential decline. Following the oral administration of the SR tablets, the absorption rate of donepezil decreased consistently with the decreased in vitro dissolution rates, represented by increased T_max_. The peak plasma concentration, C_max,_ decreased from 3.19 ± 0.67 ng/mL for the IR tablet, and to 0.78 ± 0.43 ng/mL for the SR-3 tablets, which have the slowest dissolution rate. In parallel, reduced AUC_last_ and AUC_inf_ were also observed after donepezil SR tablet administration. Dose-adjusted C_max_ and AUC values also decreased with the decrease of dissolution rates from SR-1 to SR-3. However, statistical significance in pharmacokinetic parameters among formulations was only observed for T_max_, probably due to the limited number of subjects. The relative bioavailability was 74.30% for SR-1, 59.87% for SR-2, and 40.46% for SR-3. On the other hand, the elimination half-life (t_1/2_) was comparable among IR and SR tablets.

### 3.3. Establishment of Level A IVIVC via Extended DoE-IVIVC Model

#### 3.3.1. Development of Population PK Model

**Figure 4:**
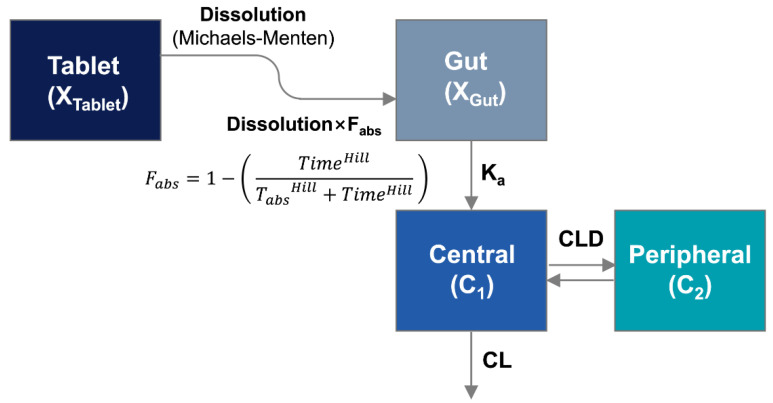
The structural model for the pharmacokinetics of donepezil in Beagle dogs.

By simultaneously fitting the observed plasma concentration vs. time data after the donepezil SR tablets, a population PK model was developed. The structure of the population PK model of donepezil is depicted in Figure 4. Similar to in vitro dissolution, in vivo dissolution of donepezil from SR tablets was described by Michaelis–Menten kinetics. The absorption of the dissolved donepezil into the systemic circulation was described by first-order kinetics. The systemic disposition of donepezil was characterized by a two-compartment model. Since the oral bioavailability of donepezil reduced as the dissolution rate decreased, the time-dependent absorption kinetics were applied to the model as described previously [4,11,15,16]. The time-dependent fraction capable of absorption (F_abs_) was multiplied by the in vivo dissolution rate to represent the decrease in the amount of donepezil absorbed into the gut compartment. 

**Figure 5:**
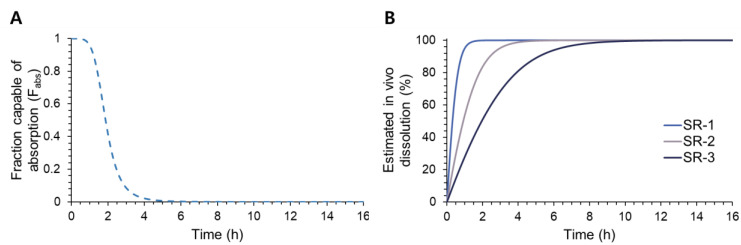
Changes in the fraction capable of absorption (F_abs_) of donepezil (**A**) and the in vivo dissolution profiles of SR-1, SR-2, and SR-3 tablets (**B**).

The developed population PK model adequately described the overall plasma concentration-time profiles. The population pharmacokinetic parameter estimates are listed in Table 4. The estimates of the in vivo dissolution rate, V_max·in vivo,_ was 3.658 h^−1^ for SR-1, 1.417 h^−1^ for SR-2, and 0.476 h^−1^ for SR-3, indicating the fast, medium, and slow dissolution rates, respectively. The population PK model-estimated F_abs_ of donepezil and the in vivo dissolution patterns of SR-1, SR-2, and SR-3 tablets are shown in Figure 5. The fraction capable of absorption in the gastrointestinal tract (F_abs_) was estimated to be complete initially but decreased sharply and became almost zero at 8 h after dosing (Figure 5A). 

#### 3.3.2. Development of DoE-IVIVC Model

Both in vitro and in vivo dissolution profiles of donepezil were described by the Michaelis–Menten kinetics. The average estimates of V_max·in vivo_, i.e., 3.658, 1.417, and 0.476 h^−1^, were much faster than V_max·in vitro_, i.e., 1.11, 0.5, and 0.334 h^−1^, for SR-1, SR-2, and SR-3, respectively. By linear regression, the equation describing the correlation between V_max·in vitro_ and V_max·in vivo_ was obtained as follows:

(9) $$V_{\max\cdot \mathrm{in}\;\mathrm{vivo}}=-0.8658+4.1728\cdot V_{\max\cdot \mathrm{in vitro}}$$

**Figure 6:**
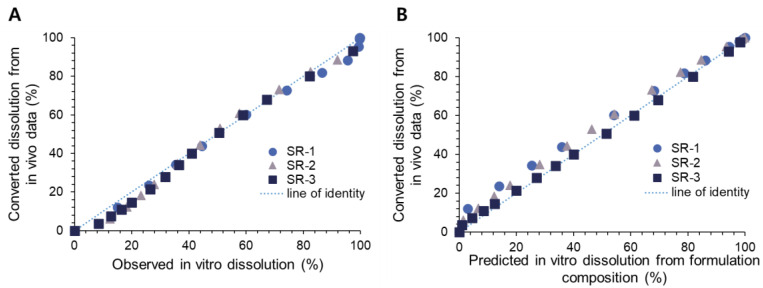
The correlation between the converted in vitro dissolution from in vivo data with the observed in vitro dissolution (**A**) and with the predicted in vitro dissolution from formulation compositions (**B**).

By using this equation (R^2^ = 0.9863), V_max·in vivo_ could be converted to V_max·in vitro,_ and in vitro dissolution profiles could be predicted. The converted dissolution from the in vivo data was correlated with the dissolution observed in vitro (Figure 6A). The converted in vitro dissolution from the in vivo data was also correlated with the DoE model-predicted in vitro dissolution from the formulation compositions (Figure 6B). The estimated in vitro dissolution from the in vivo data correlated well with both the observed and DoE model-predicted in vitro dissolution, indicating the establishment level A IVIVC.

**Figure 7:**
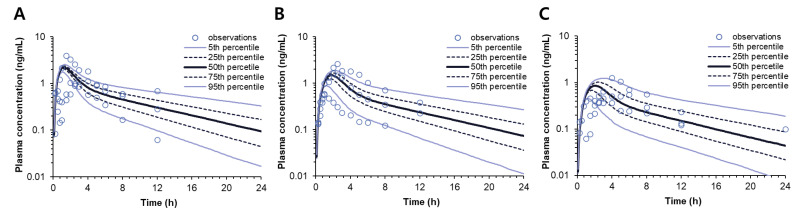
A comparison of the final extended DoE-IVIVC model-predicted and observed plasma concentration vs. time profiles following the oral administration of SR-1 (**A**), SR-2 (**B**), and SR-3 (**C**) donepezil SR tablets in Beagle dogs.

Finally, the DoE and IVIVC equations were applied to the population pharmacokinetic model to develop the DoE-IVIVC model. The final DoE-IVIVC model allowed the prediction of in vivo pharmacokinetic profiles after oral administration in Beagle dogs by using the formulation of each SR tablet as an input (Figure 7). Table 5 shows the predicted C_max_ and AUC via the DoE-IVIVC model from their formulation compositions. The absolute PE% ranged from 6.84 to 10.30% for C_max_ and from 0.98 to 5.19% for AUC. The mean PE% was 8.91% and 3.27% for C_max_ and AUC, respectively. According to the FDA guidance, the predictability of conventional IVIVC is considered acceptable when the PE% for C_max_ and AUC is lower than 15% for each formulation and the mean PE% values are lower than 10% [1]. 

## 4. Discussion

The potential utility of the extended DoE-IVIVC model to evaluate the impact of formulation changes on the pharmacokinetics of the SR tablets has been examined by using donepezil as a model drug. The extended DoE-IVIVC model integrated DoE and population pharmacokinetic model-based IVIVC to interlink the formulation, in vitro dissolution, and in vivo pharmacokinetic profiles. DoE and IVIVC have been separately pursued to facilitate the different design and development stages of new SR formulations. By combining the two approaches into a population pharmacokinetic model, the extended DoE-IVIVC model integrates the entire formulation development process and allows for predicting in vivo pharmacokinetics of an SR tablet from its formulation composition. 

The application of IVIVC to the initial formulation design process has been pursued previously for hydrophilic matrix SR tablets. The model was applied to a series of SR tablets by varying one formulation factor, HPMC contents (%), leading to different drug-release rates [4]. By establishing IVIVC, the in vivo plasma concentration-time profile of an SR tablet of baclofen could be predicted from its HPMC content [4]. The potential of the physiologically based pharmacokinetics (PBPK) model to design formulations for an IVIVC study by using PBPK-predicted in vivo drug exposure for the formulation design space has also been introduced [2]. The integrated use of DoE and IVIVC has recently been proposed to predict the in vivo pharmacokinetics by the changes of the entire formulation composition and vice versa [5]. The present study demonstrated the utility of the extended DoE-IVIVC model to predict in vivo pharmacokinetics from the formulation compositions of SR tablets by using donepezil as a model drug. The developed extended DoE-IVIVC model successfully provided the predictions of in vivo pharmacokinetics from the formulation compositions of donepezil SR formulations.

The drug release kinetics from donepezil SR tablets were characterized by the Michaelis–Menten model. The maximum rate of dissolution, V_max_, has been utilized as a representing dissolution parameter in both DoE and IVIVC. Although there are other more mechanistic models available to describe and predict the drug release kinetics [17,18], the Michaelis–Menten model could successfully describe the current dissolution profiles of donepezil SR tablets. Thus, a mathematical relationship between the formulation factors and the in vitro dissolution parameter, i.e., V_max·in vitro_, was defined via DoE. The formulation compositions of 14 SR tablets were designed by the mixture design and provided the independent factors for the DoE equation. After the in vitro dissolution profiles of SR tablets were obtained, the dissolution parameters, V_max·in vitro_, were estimated, which were used as responses to derive the DoE equation. The derived DoE model could accurately predict the V_max·in vitro_ of SR tablets from their formulation compositions (Table 2), which in turn allowed one to predict the entire dissolution profiles of each SR tablet (Figure 2). 

The pharmacokinetic model was developed to characterize the plasma concentration-time profiles of donepezil after the oral administration of SR tablets. Our data showed that the dose-normalized C_max_ values of the donepezil SR tablets generally decreased compared with that of the IR tablet, indicating that the dissolution of donepezil from SR tablets was prolonged in the gastrointestinal tract. Interestingly, the bioavailability of the donepezil SR tablets became lower, and their CL/F was higher than those of the reference IR tablet (Table 3). The lower bioavailability of the SR tablets indicates the potential presence of a narrow absorption window for the drug in the gastrointestinal tract [11,15]. Therefore, to describe the reduced bioavailability of this drug for the SR formulations, possibly due to the presence of an absorption window of the drug, the time-dependent absorbed fraction was applied to the model. Based on the population pharmacokinetic model, the in vivo fraction capable of absorption was estimated to decrease over time, and the in vivo dissolution profiles of donepezil were estimated (Figure 5).

The developed pharmacokinetic model could successfully characterize all the pharmacokinetic profiles and provide the estimates of the pharmacokinetic parameters, including the in vivo dissolution parameter, V_max·in vivo,_ for each SR tablet (Table 4). By correlating the in vitro and in vivo dissolution parameters, i.e., V_max·in vitro_ and V_max·in vivo_, an IVIVC equation was derived, which allowed interconversion between the in vitro dissolution and in vivo dissolution. Finally, the two model equations, i.e., DoE and IVIVC, were integrated into the pharmacokinetic model to compose the extended DoE-IVIVC model that connected the formulation composition, in vitro dissolution, and in vivo pharmacokinetics. Thus, in the extended DoE-IVIVC model, the formulation compositions provided the input to predict V_max·in vitro,_ which was, in turn, converted to V_max·in vivo_ and used to simulate the entire plasma concentration-time profile of each SR tablet (Figure 7). The prediction error (PE%) for C_max_ and AUC with the formulation changes was less than 10.3% and 5.19%, respectively (Table 5), which sufficiently satisfied the criteria of conventional IVIVC [1]. 

Overall, this study provides a validation of the extended DoE-IVIVC model as a new platform to predict the in vivo pharmacokinetics of SR tablets from their formulation composition for donepezil. Based on the present extended DoE-IVIVC model, the in vivo pharmacokinetic performance of a new formulation could be predicted in the initial stage of development from the formulation compositions. It is also possible to design a formulation to obtain target pharmacokinetic profiles. Therefore, the present approach would minimize trials and errors during the development process of new formulations and eventually improve the success rates. 

The utility of the current extended DoE-IVIVC model might be further evaluated to predict pharmacokinetics in humans by using clinical data. Since the data set for this study has been generated in Beagle dogs, the prediction is limited to the same animal species. Predicting human pharmacokinetics via the present modeling approach is only possible if the in vivo pharmacokinetics data are obtained in humans. The PBPK model has a distinctive advantage for interspecies prediction. Thus, the prediction of human pharmacokinetics of drug formulations from animal data has also been investigated by adopting PBPK modeling [19,20], which might be of interest for future studies to extend the present results further. The application of the present modeling approaches to develop other forms of drug formulations than oral SR tablets may also be of interest for future studies.

## Figures and Tables

**Table 1:** The experimental design for donepezil SR formulations and the observed dissolution parameters.

Run Order	Fixed Factors		Control Factors			Response Factor
	Donepezil Hydrochloride (%)	Magnesium Stearate (%)	x_1_	x_2_	x_3_	y
			Lactose (%)	HPMC 100 cps (%)	HPMC 4000 cps (%)	1/V_max·in vitro_ (h)
1	3.910	0.990	80	0	15.1	1.326
2	3.910	0.990	72.5	15.075	7.525	1.538
3	3.910	0.990	72.5	7.525	15.075	1.887
4	3.910	0.990	50	0	45.1	3.448
5	3.910	0.990	80	7.55	7.55	1.136
6	3.910	0.990	57.5	30.075	7.525	1.961
7	3.910	0.990	50	22.55	22.55	2.198
8	3.910	0.990	57.5	7.525	30.075	2.941
9	3.910	0.990	65	0	30.1	2.778
10	3.910	0.990	65	30.1	0	1.528
11	3.910	0.990	65	15.05	15.05	2.273
12	3.910	0.990	50	45.1	0	1.796
13	3.910	0.990	80	15.1	0	0.729
14	3.910	0.990	65	15.05	15.05	2.222

**Table 2:** The composition (*w*/*w*%) of selected donepezil SR tablets and a comparison between model-predicted and observed response factors for external validation.

No.	Fixed Factors		Control Factors			Response Factor		
	Donepezil Hydrochloride (%)	Magnesium Stearate (%)	Lactose (%)	HPMC 100 cps (%)	HPMC 4000 cps (%)	1/V_max·in vitro_ (h)		
						Predicted	Observed	(PE%)
SR-1	3.910	0.990	78.00	15.90	1.20	0.917	0.901	(1.78%)
SR-2	3.910	0.990	69.00	13.90	12.20	1.985	2.000	(0.56%)
SR-3	3.910	0.990	54.00	9.90	31.20	2.889	2.994	(3.50%)
SR-4	3.910	0.990	63.00	24.00	8.10	1.940	2.000	(2.97%)
SR-5	3.910	0.990	72.00	8.80	14.30	1.936	2.000	(3.20%)

**Table 3:** The non-compartmental pharmacokinetic parameters (mean ± S.D.) of donepezil after the oral administration of donepezil IR and SR formulations in Beagle dogs.

Parameters	IR (*n* = 3)	SR-1 (*n* = 3)	SR-2 (*n* = 3)	SR-3 (*n* = 3)
Dose (mg)	4.562	5	5	5
t_1/2_ (h)	5.39 ± 2.02	4.12 ± 1.55	7.37 ± 3.71	7.16 ± 6.72
T_max_ (h) ^a^	1.5 (1.0–1.5)	2.0 (1.5–3.0)	2.0 (1.0–2.5)	4.0 (2.5–6.0) *
C_max_ (ng/mL)	3.19 ± 0.67	2.06 ± 1.62	1.62 ± 1.01	0.78 ± 0.43
AUC_last_ (ng·h/mL)	11.08 ± 2.29	9.03 ± 5.25	7.27 ± 5.37	4.91 ± 1.53
AUC_inf_ (ng·h/mL)	13.29 ± 2.96	11.52 ± 7.93	9.46 ± 5.59	6.21 ± 1.82
V_z_/F (L)	2679.87 ± 806.50	3048.69 ± 834.81	9291.73 ± 10,208.28	8361.11 ± 7150.01
CL/F (mL/min)	5941.79 ± 1467.11	10,210.53 ± 6621.98	11,840.78 ± 8144.75	14,453.44 ± 4770.17
MRT (h) ^b^	6.55 ± 1.73	6.95 ± 2.14	9.76 ± 3.88	11.73 ± 7.46
Relative bioavailability (%)	-	74.30%	59.87%	40.46%

^a^ Data are presented as the median (minimum–maximum); ^b^ mean residence time; and * *p* < 0.05 vs. IR.

**Table 4:** The population pharmacokinetic parameter estimates of donepezil.

Parameter	Symbol	Unit	Population Mean	BSV ^a^ (%)
V_d_ of the central compartment	V_1_	L	1124.69	2.78
V_d_ of the peripheral compartment	V_2_	L	1850.80	2.58
Systemic clearance	CL	L/h	388.07	41.03
Distribution clearance	CL_D_	L/h	550.90	10.61
1st order rate constant of drug absorption	K_a_	1/h	2.21	4.33
Time associated with the maximal changes in F_abs_	T_abs_	1/h	1.87	62.53
Drug amount at which the dissolution rate is half of V_max_	AM_50·in vivo_	-	0.90	NA *
Hill coefficient	γ	-	5	NA *
V_max·in vivo_ for SR-1	V_max·in vivo·SR-1_	1/h	3.658	5.80
V_max·in vivo_ for SR-2	V_max·in vivo·SR-2_	1/h	1.417	4.24
V_max·in vivo_ for SR-3	V_max·in vivo·SR-3_	1/h	0.476	3.86

^a^ BSV, between subject variability; * NA, not applicable.

**Table 5:** The absolute percentages of the prediction error (PE) for C_max_ and AUC of SR-1, SR-2, and SR-3 by the extended DoE-IVIVC model.

Formulation	C_max_ (ng/mL)			AUC (ng·h/mL)		
	Observed (*n* = 3)	Predicted	PE (%)	Observed (*n* = 3)	Predicted	PE (%)
SR-1	2.06 ± 1.62	2.20	6.84	9.03 ± 5.25	9.50	5.19
SR-2	1.62 ± 1.01	1.45	10.30	7.27 ± 5.37	7.34	0.98
SR-3	0.78 ± 1.53	0.85	9.59	4.91 ± 1.53	4.73	3.63

## Data Availability

The data presented in this study are available on request from the corresponding author.
